# Effects of Home-Based Baduanjin Exercise on Left Ventricular Remodeling in Patients With Acute Anterior ST-Segment Elevation Myocardial Infarction: Study Protocol for a Randomized Controlled Trial

**DOI:** 10.3389/fcvm.2022.778583

**Published:** 2022-02-09

**Authors:** Yinhe Cai, Liang Kang, Haiyi Li, Yuan Luo, Junmao Wen, Zhaohui Gong, Qingmin Chu, Yijun Qiu, Chuanjin Luo, Keyu Chen, Xinjun Zhao, Rong Li

**Affiliations:** ^1^The First Clinical Medical College of Guangzhou University of Chinese Medicine, Guangzhou, China; ^2^Department of Cardiovascular Medicine, The First Affiliated Hospital of Guangzhou University of Chinese Medicine, Guangzhou, China; ^3^Department of Interventional Room, The First Hospital Affiliated of Guangzhou University of Chinese Medicine, Guangzhou, China

**Keywords:** home-based, Baduanjin exercise, left ventricular remodeling, anterior STEMI, clinical trial

## Abstract

**Background:**

Left ventricular (LV) remodeling after ST-segment elevation myocardial infarction (STEMI) is a major pathological basis associated with heart failure and increased mortality. Exercise-based cardiac rehabilitation has been verified to significantly improve prognosis and quality of life. As a traditional Chinese Qigong, Baduanjin exercise has effectively alleviated adverse LV remodeling in STEMI patients. Despite this, participation in exercise rehabilitation remains low, and home-based exercise rehabilitation may be an alternative approach. Besides, anterior STEMI is reported to have higher risk of adverse LV remodeling. However, the efficiency regarding home-based Baduanjin exercise on LV remodeling in anterior STEMI patients remains uncertain currently.

**Methods/Design:**

A single-blind, randomized controlled clinical trial was conducted to explore the efficacy and safety of home-based Baduanjin exercise in anterior STEMI patients compared with moderate intensity aerobic walking. A total of 114 participants were assigned randomly to the Baduanjin group or walking control group at a 1:1 ratio. Eligible participants practiced Baduanjin or walking exercise (5 times a week) for 12 weeks, and then followed up for another 12 weeks. The primary outcome is a relative change in the LV end-diastolic volume. The secondary outcomes include the plasma levels of hypersensitive C-reactive protein and interleukin 6, health-related quality of life measured by EQ-5D-5L, LV ejection fraction, patient health questionnaire-9, generalized anxiety disorder screener-7, short physical performance battery score, and clinical endpoint events. The proportion of circulating regulatory T-cells were also assessed. Adverse events were recorded throughout the trial for safety evaluation. Data were be analyzed by researchers blinded to the treatment allocation.

**Discussion:**

This study provided powerful evidence for the use of home-based Baduanjin exercise in anterior STEMI patients in alleviating LV remodeling and improving clinical outcomes.

**Trial Registration:**

The Research Ethics Committee of the First Affiliated Hospital of Guangzhou University of Chinese Medicine has approved this study (ZYYECK[2020]045). Written informed consent of patients were required. This trial is registered in the Chinese Clinical Trial Registry (ChiCTR2100047298).

**Dissemination:**

Our results will be published in peer-reviewed journals and disseminated through academic conferences and the Internet.

## Introduction

Acute myocardial infarction (AMI) is a cardiac emergency with substantial morbidity and mortality (1); AMI survivors are prone to cardiac insufficiency and unfavorable prognosis (2). The extent of left ventricular (LV) remodeling has been reported to be the major pathological basis associated with the prognosis after AMI (3). Multiple published studies (4, 5) have demonstrated that cardiac rehabilitation (CR), especially exercise-based CR, can significantly improve the prognosis and quality of life in patients with AMI (6, 7). Moreover, increasing studies (8, 9) have shown that exercise-based CR can reverse LV remodeling in patients with AMI by decreasing LV end-diastolic volume (LVEDV) or LV end-systolic volume (LVESV) and increasing LV ejection fraction (LVEF).

Despite this, current participation rates in exercise-based rehabilitation are generally extremely low, whether in developed or developing countries. Reported barriers to attendance involve inconvenient transportation, high out of pocket expenses, scheduling conflict, and lack of professional rehabilitation center and facility (10). Notably, home-based exercise rehabilitation may be a potential solution to these barriers. Some research has found that home-based CR is not inferior to a center-based CR program both in terms of efficacy and safety (11). The latest work has demonstrated that patients participating in home-based exercise rehabilitation make favorable improvement in exercise capacity, quality of life, and adherence (12, 13). Meanwhile, the incidence of cardiac events such as mortality, coronary revascularization, and hospital readmissions are also decreased. Therefore, home-based exercise rehabilitation is worth generalizing to patients with AMI.

Baduanjin exercise, as a traditional Chinese Qigong exercise designed to enhance health, is a moderate intensity aerobic exercise that can be practiced easily and performed at any place without restrictions (14). To some extent, many clinical studies have manifested that Baduanjin exercise exerts a positive impact on enhancing cardiopulmonary exercise function (15, 16), protecting vascular endothelial function, alleviating dyslipidemia, and stabilizing glucose and blood pressure (17, 18). A recent study (19) has indicated that a 12-week Baduanjin center-based CR program could effectively alleviate adverse LV remodeling in patients surviving ST-elevation acute myocardial infarction (STEMI). However, the effect of home-based Baduanjin exercise is still unclear, and the distinction among STEMI patients with different infarction location is unexplored in their study. Anterior STEMI, reported to be an independent predictor, is generally accepted to have higher risk of adverse LV remodeling than other location (20). Therefore, it is valuable to explore the efficiency of home-based Baduanjin exercise on LV remodeling and clinical outcomes in anterior STEMI patients.

To date increasing evidences (21) have shown that adaptive immune response is involved in post-ischemic cardiac remodeling after MI, and among them, T cells play a crucial role in the process of impairment and healing in the infarcted heart. Latest studies (22, 23) have indicated that regulatory T cells (Tregs) may serve as emerging targets for cardiac tissue repair as they can terminate the proinflammatory phase and produce antiinflammatory cytokines [e.g., interleukin (IL)-10 and transforming growth factor beta (TGF-β)] at the site of tissue injury. Currently, accumulative evidences (24, 25) have elucidated that regular exercise could make a difference in increasing the numbers and proportions of circulating antiinflammatory Tregs. Therefore, it is necessary to explore whether the possible mechanism of Baduanjin exercise on LV remodeling is related to *T*-cells.

Against this backdrop, we designed a randomized controlled clinical trial to compare the efficacy and safety of 12-week home-based Baduanjin exercise with moderate intensity aerobic walking in patients with anterior STEMI. Patients with a short physical performance battery (SPPB) score more than 9, which are considered to have an exceptional functional capacity, were included to reduce variability among different patients. We assume that home-based Baduanjin exercise is better than moderate intensity aerobic walking in alleviating LV remodeling and improving clinical outcomes such as health-related quality of life (HRQoL) and clinical endpoint events. Flow cytometry was used to detect the changes of T lymphocyte subsets, which can help us to analyze the possible mechanism of home-based Baduanjin exercise in inhibiting LV remodeling after anterior STEMI.

## Methods and Analyses

### Design and Settings

**Figure 1:**
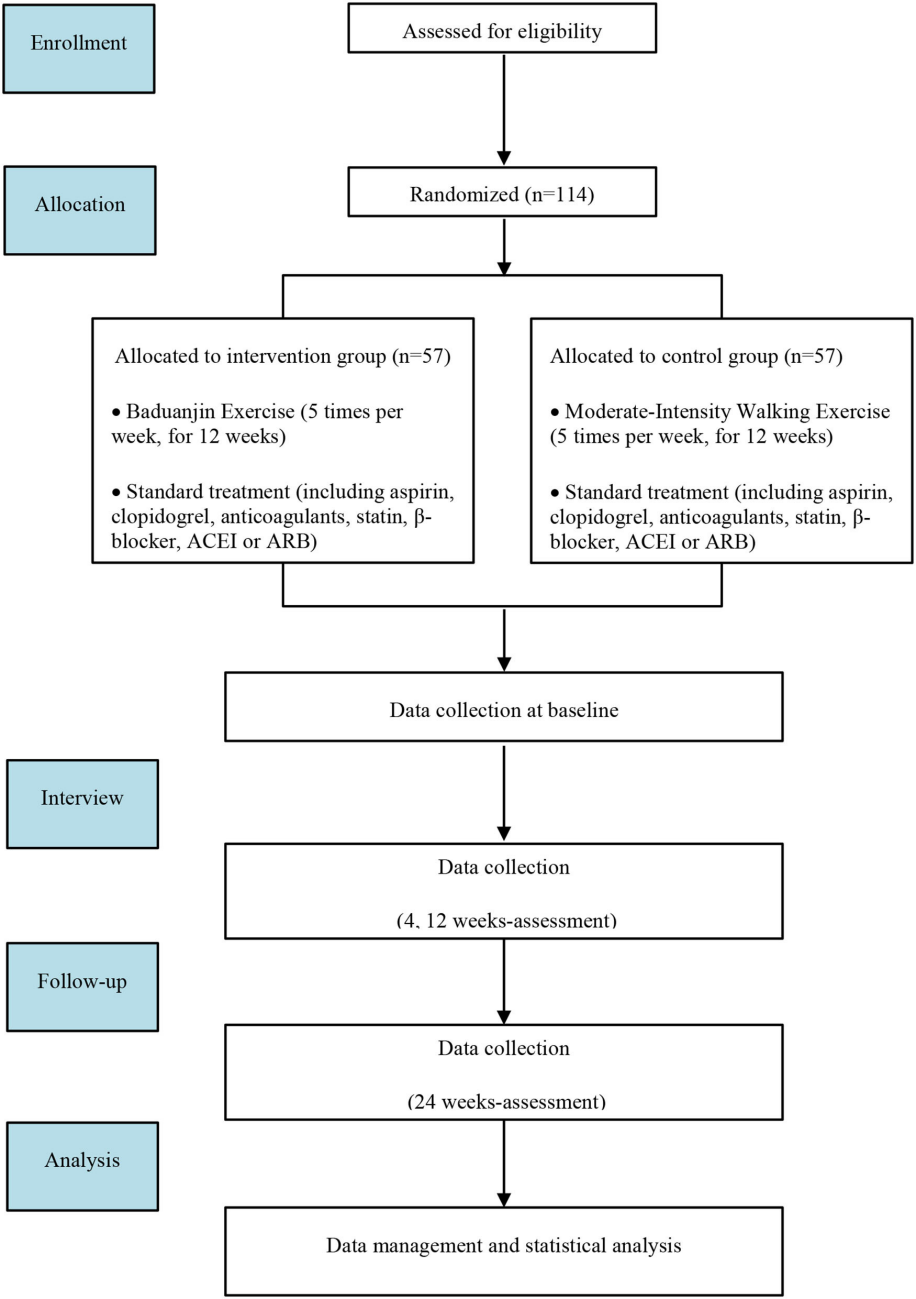
Flowchart of the clinical trial design. The template is from the CONSORT 2010 flowchart. ACEI, angiotensin-converting enzyme inhibitor; ARB, angiotensin receptor blocker.

This study is a single-center, open, randomized controlled trial that was conducted at The First Affiliated Hospital of Guangzhou University of Chinese Medicine. A total of 114 participants were recruited after providing written informed consent. The participants were randomly assigned in a 1:1 ratio to either the Baduanjin exercise group or the walking control group. This trial consisted of a 12-week intervention period and a 12-week follow-up period. A flowchart of the trial is illustrated in Figure 1.

This trial protocol has been approved by the Ethics Committee of The First Affiliated Hospital of Guangzhou University of Chinese Medicine (project number: ZYYECK[2020]045) and registered in China Clinical Trial Registry (ChiCTR2100047298).

### Recruitment

Participants were recruited from The First Affiliated Hospital of Guangzhou University of Chinese Medicine. The potential patients were informed about the information of this trial, including the objectives, process, intervention methods, potential adverse effects, and advantages. Each eligible participant was asked sign the informed consent.

### Study Population

#### Inclusion Criteria

(1) Men or women, between 18 and 75 years old.
(2) First anterior STEMI was diagnosed based on typical chest pain, cardiac enzyme elevation, and persistent st-segment elevation of 2 mm in 2 or more contiguous anterior leads or 4 mm total st-segment deviation sum in the anterior leads;
(3) Received successful primary percutaneous coronary intervention (PCI) within 12 h after chest pain onset.
(4) The SPPB score more than 9 at the inclusion visit before discharge.
(5) Volunteer, understand, and provide written informed consent.

#### Exclusion Criteria

(1) Plan to have cardiac surgery or elective multivessel PCI during the trial.
(2) Patients with prior MI or coronary artery bypass grafting surgery.
(3) Patients with myocardial ischemia at rest or on exertion, cardiogenic shock, severe heart failure (LVEF <35%), uncontrollable malignant arrhythmia, severe chronic obstructive pulmonary disease, or severe valvular disease necessitating surgery.
(4) Uncontrolled hypertension, systolic blood pressure (SBP) ≥180 mmHg, and/or diastolic blood pressure (DBP) ≥110 mmHg; or SBP <90 mmHg and/or DBP <50 mmHg.
(5) Combined with psychiatric manifestations or malignancy.
(6) Expectation life <1 year.
(7) Participation in other clinical exercise studies within 3 months.
(8) Patients who are unable to exercise due to physical disabilities or other reasons.
(9) Researcher's estimate that the patient may be unable to complete the study

#### Criteria for Withdrawal and Removal

(1) Serious comorbidities, serious complications, or serious adverse events (AEs) during the study, such as repeated hospitalization for MI or heart failure;
(2) Receive adjunctive treatments without permission, which will affect the assessment of efficacy and safety.
(3) Poor compliance by participants, or the amount of exercise does not meet the regulations (<80% or >120%);
(4) Participants request to withdraw from the study halfway;
(5) Researchers determine it is not safe to continue participating in the trial.

### Randomization and Blinding

Participants were randomized to the Baduanjin exercise group or the walking control group in a 1:1 ratio using a table of random numbers, which is generated in the R statistical package. The grouping information was retained in an opaque envelope and sealed subsequently. Participants were recruited in a natural, unpredictable order. After screening, the study coordinator opened an envelope sequentially to obtain a random number and grouping information for each included participant.

Given the complete difference of the exercise interventions, it is not possible to blind study participants and exercise instructors throughout the entire duration of this trial. However, the staff performing the echocardiography, technicians in clinical laboratory, investigators conducting the outcomes assessments, and statistical analysis were blinded to the assignment.

### Study Intervention

Home-based CR were adopted for both the groups. Eligible participants were allocated randomly to the Baduanjin group or the walking control group, and individualized exercise prescription was formulated.

Both groups exercised at least 5 times per week for 12 weeks at home. Exercise intensity was monitored using Borg's rating of perceived exertion (RPE) scale, or regulated based on HRmax determined during the initial 6-min walk test. Exercise sessions in both groups included a warm up, movement, and relaxation period. Moreover, a movement diary was provided to all the participants to record their daily exercise activity for the duration of the trial. At the end of the 12-week mark, patients were encouraged to continue their exercise training at home.

#### Baduanjin Exercise Group

The Baduanjin exercise group practiced Baduanjin at home. A professional coach guided the training of participants during hospitalization and supervised them throughout the entire intervention period. A standardized Baduanjin exercise protocol was formulated following the “Healthy Qigong Baduanjin” published by the General Administration of Sports in 2003 (26). Baduanjin exercise contains eight postures, and each training lasts about 12 min. Each routine session lasted for 30 min at least (including 5 min of warm up, 2 or 3 times of Baduanjin taring, and 5 min of relaxation), 5 times per week for 12 weeks. The exercise intensity and energy expenditure was a constant at Borg's RPE 12–13 scale or 55–75% of the peak HR. Regularly reviewing video recordings and timely feedback of the subjects was done to ensure participants compliance and accurate performance of each movement. All participants were instructed to maintain their usual activities and refrain from new strength training.

#### Control Group

The walking control group practiced moderate intensity walking exercise on a treadmill or outdoors at a frequency of 5 times per week, over a 12-week period. Each routine session contained three components, 5 min of warm up, at least 20-min walking training, and 5 min of relaxation. The exercise intensity and energy expenditure was a constant at Borg's RPE 12–13 scale or 55–75% of the peak HR. All participants were instructed to maintain their usual activities and refrain from new strength training.

### Concomitant Treatment

All participants received standard treatment, such as aspirin, clopidogrel, ticagrelor, anticoagulants, statin, beta-receptor blocker, angiotensin-converting enzyme inhibitor (ACEI), or angiotensin receptor blocker (ARB). Any medical therapy change was recorded in a combined medication record form.

### Outcomes

#### Primary and Secondary Outcomes

Table 1 illustrates the items to be measured and time window of data collection in detail. The primary outcome is LV remodeling index measured by transthoracic echocardiography (relative change in LVEDV seen at 12-week and 24-week follow-up compared with baseline during admission), representing structural changes after anterior STEMI. The presence of LV remodeling is defined as ≥20% increase in LVEDV at any time during the first 12 months post-STEMI (27).

**Table 1:** Study schedule.

**Study phase time**	**Baseline period**	**Intervention period**		**Follow-up**
	**Visit 1**	**Visit 2**	**Visit 3**	**Visit 4**
	**-7 to 0 days**	**4 weeks**	**12 weeks**	**24 weeks**
**Data collection at baseline**				
Informed consent	×			
Inclusion/exclusion criteria	×			
Allocation	×			
Demographic data	×			
Previous history, medical history, and allergies	×			
Comorbidities and co-medications	×			
**Intervention**				
Baduanjin Exercise				
Moderate-Intensity Walking Exercise				
**Safety evaluation**				
Vital signs	×	×	×	
Physical examination	×	×	×	
Blood routine	×	×	×	
Liver and kidney function	×	×		
Electrocardiograph	×	×	×	
**Efficiency evaluation**				
Echocardiographic parameters	×		×	×
Health-Related Quality of Life	×	×	×	
Inflammatory cytokines of hs-CRP, IL-6	×		×	
Depression and anxiety	×	×	×	
Short physical performance battery	×	×	×	
Clinical-endpoint events		×	×	×
**Other work**				
Flow Cytometry Analysis of T Lymphocytes	×		×	
Record AEs		×	×	×
Complications due to exercise		×	×	
Evaluate compliance		×	×	×

The secondary outcomes are

(1) Improvement in HRQoL. This was assessed according to EQ-5D-5L. The EQ-5D is a simple, generic HRQoL instrument that is widely used as a patient-reported outcome measure. EQ-5D-5L consists of utility index (UI) and visual analog score (VAS). The UI is calculated from patient scoring of five dimensions (mobility, self-care, usual activities, pain/discomfort, anxiety/depression). The EQ-5D-5L UI in Chinese is between −0.391 and 1.00 (28).
(2) Improvement in echocardiographic measurements of LVEF calculated with Simpson's method from 2-dimensional, apical, 2-chamber, and 4-chamber views (29).
(3) Changes in plasma levels of inflammatory cytokine hypersensitive C-reactive protein (hs-CRP) and IL-6.
(4) Changes in patient health questionnaire-9 (PHQ-9) and generalized anxiety disorder screener-7 (GAD-7) score.
(5) Changes in SPPB score.
(6) The incidence rate of clinical endpoint events (cardiovascular events, cardiogenic death, and all-cause death).

#### Safety Outcomes

Safety outcomes comprise vital signs (body temperature, heart rate, breathing, and blood pressure), electrocardiogram, laboratory examinations (routine blood test, liver, and kidney function examination), and AEs (which were recorded throughout the trial). AEs may include medication-withdrawal AEs (e.g., dizziness, headache, pain, fatigue, loss of strength, breathlessness, somnolence, and sleep difficulties), exercise injury, and fall (30).

### Flow Cytometry Analysis of T Lymphocytes

The numbers and proportions of adaptive immune status focused on T cells were assessed by flow cytometry analysis at the baseline and at the end of the intervention (12 weeks), including lymphocytes, T-cells (CD3+), and the T-cells subpopulations such as cytotoxic T-cells (CD3+CD8+) and T-helper cells (CD3+CD4+). Additionally, the proportions of Tregs (CD3+CD4+CD25+) was also assessed. Most studies have concluded that adaptive immune response focused on *T*-cells exert a significant role to regulate inflammation and repair after myocardial infarction. In particular, depletion of Tregs exacerbate cardiac inflammation and LV remodeling (31), while their increase (32) or adoptive transfer (33) contributes to heart healing post-MI. Therefore, Tregs can be a potential solution for cardiac tissue repair (34, 35).

### Sample Size

The sample size is calculated based on the expected improvement in LVEDV between the Baduanjin exercise group and the walking control group. Referring to clinical studies (19, 36), we assumed that the improvement in LVEDV is 5 ml, and the combined standard deviation (SD) is 10 ml in the present study. On the basis of a rate of type-I error of α = 0.05, a power of 80% (rate of type-II error of β = 0.2), and considering a possible dropout rate of 10%, the experimental and control groups each required at least 57 patients.


$$\text{n}1\;=\;{\left[\frac{\left(\mu_{\alpha }\;+\;\mu_{\beta }\right)\sigma }{\delta }\right]}^{2}\;\frac{\left(1\;+\;\kappa \right)}{\kappa },\;{\text{n}}_{2}\;=\;\kappa {\text{n}}_{1}$$

In this formula, κ is the ratio between the two sample cases, δ is the expected improvement in LVEDV, and σ is the combined SD.

### Statistical Analyses

An intention-to-treat analysis was conducted. Moreover, data was also analyzed as per the CONSORT statement. Statistical analysis was conducted by third-party statisticians, who were blinded to the group allocation and intervention process, using statistical software package SPSS 23.0 (SPSS Inc., Chicago, USA). Regarding categorical variables, these were described as counts or percentages. Continuous variables with normal distribution were displayed as mean ± SD, whereas data with non-normal distribution was displayed as median and interquartile range. The chi-square test or Fisher's exact probability method was applied for categorical data, whereas the Student's *t*-test was applied for continuous data with a normal distribution. Homogeneity test of variance was conducted among the groups, with a cutoff level of 0.05, and the corrected *t*-test was used when the variance was uneven. If the data does not follow a normal distribution, the Mann-Whitney U test was performed to analyze intragroup or intergroup differences before and after treatment, respectively. All tests were two-sided, and a *P* value < 0.05 was considered statistically significant. The method of analysis for specific outcomes is shown in Table 2.

**Table 2:** Outcomes and methods of analyses.

**Outcome/variable**	**Measures**	**Methods of analyses**
**Baseline balance test**	Continuous variables (age, BMI, heart rate, blood pressure, symptom-to-balloon time, number of stents, etc.)	*t*-text/Mann-Whitney U text
	Categorical variables (gender, comorbidities, discharge medications, infarct-related artery, etc.)	Chi-squared test/Fisher's exact test /rank-sum test
**Primary outcome**		
Echocardiographic parameters	LVEDV, LVESV and LVEF	*t*-text/Mann-Whitney U text
**Secondary outcomes**		
HRQoL	EQ-5D-5L utility index and VAS	*t*-text/Mann-Whitney U text
Plasma levels of hs-CRP, IL-6		*t*-text/Mann-Whitney U text
Depression and anxiety	PHQ-9 and GAD-7	*t*-text/Mann-Whitney U text
SPPB		*t*-text/Mann-Whitney U text
Clinical-endpoint events	Clinical-endpoint event rate	Chi-squared test/Fisher's exact test
**Safety outcomes**		
AEs	Percent and cases of AEs	Chi-squared test/Fisher's exact test

*LVEF, Left ventricular Ejection Fraction; LVEDV, Left Ventricular End-Diastolic Volume; LVESV, Left Ventricular End-Systolic Volume; SPPB, Short Physical Performance Battery; HRQoL, Health-Related Quality of Life; PHQ-9, Patient Health Questionnaire-9; GAD-7, Generalized Anxiety Disorder Screener-7; AEs, Adverse Events*.

### Quality Control

Quality control throughout the entire experimental process was carried out, and a quality control committee was established, which composed of 3–4 experts from the medical technology department, and Statistical Teaching and Research Office. Prior to the trial, all researchers (research assistants, investigators, physicians, and data analyzers) received unified preclinical trial training to fully understand the trial protocol, standard study procedures, and their corresponding roles, for example, research assistants reminded participants to exercise at home and complete exercise diaries. In addition, all investigators were trained to perform outcome assessments, data management, and fill in the case report forms (CRFs) uniformly. Meanwhile, periodical meetings were organized to discuss problems during the trial and figure out the best solutions. This trial was inspected by the ethics committee, sponsor, and clinical research organization throughout the study.

### Trial Status

This is an ongoing trial. This study is recruiting participants currently.

## Discussion

This study determined the efficacy and safety of home-based Baduanjin exercise in anterior STEMI patients on LV remodeling and clinical outcomes, as well as the changes on T lymphocyte subsets detected by flow cytometry.

Acute myocardial infarction is a serious problem in the world and especially brings great physical discomfort and psychological burden to patients, with a high morbidity and mortality (37). LV remodeling after STEMI, caused by an inflammatory response and mediated by various cells (38), is generally associated with heart failure and increased mortality (39). Substantial evidences have established the value of CR, especially exercise-based CR, in patients with AMI (40). Structured exercise training, regardless of training modality, has proven to alleviate LV remodeling (41) and improve quality of life in patients post AMI (6, 7). Despite this, current participation rates in exercise-based rehabilitation are generally very low, and home-based exercise rehabilitation may be an alternative approach that could alleviate some of these barriers.

Baduanjin exercise is a moderate intensity aerobic exercise that can be learned easily and practiced at any place without limitations, which is a popular and safe community exercise in China (14). A recent study showed that a 12-week Baduanjin center-based CR program could effectively alleviate adverse LV remodeling in patients surviving STEMI (19). However, their research did not explore different efficacy of Baduanjin exercise in patients with various infarction location of MI. It is generally accepted that STEMI located in the anterior is at a much higher risk of adverse ventricular remodeling than the other location. Furthermore, considering the low participation rates of centrr-based exercise rehabilitation, it is meaningful to consider that home-based Baduanjin exercise can alleviate the negative effects of adverse LV remodeling after anterior STEMI.

Currently, the potential mechanisms of aerobic exercise training on ameliorating adverse LV remodeling are unclear and partly attributed to immune function. Strong evidences have shown that adaptive immune response focused on T cells after cardiac damage plays an important role to regulate inflammation from the acute to chronic phase, in which T lymphocytes are closely related to the process of AMI damage and repair (42). Furthermore, Tregs, one of the T cells subsets, is considered to be a potential protective factor for tissue repair to reduces loss and promote healing, which is able to transform the proinflammatory stage of local tissue to antiinflammatory or repair stage. Current studies have indicated that regular exercise is associated with increased numbers and proportions of circulating antiinflammatory Tregs (24, 25).

Therefore, this study is designed to explore the effects and safety of home-based Baduanjin exercise on LV remodeling and clinical outcomes in patients with anterior STEMI, and whether it can regulate T lymphocyte subsets, especially the proportion of Tregs. In addition, we included patients with SPPB score more than 9 to reduce variability among different patients, because they are considered to have exceptional functional capacity.

Our trial has three main limitations. First, this study is a single-center study, and compared with other multicenter comprehensive studies the sample size is smaller. It is unknown whether similar effects are obtainable in other regions and ethnic groups. Second, this trial has only been followed up for 6 months, and the follow-up time can be appropriately extended to clarify the long-term clinical results and potential effects of home-based Baduanjin exercise. Third, only patients with exceptional physical performance (SBBP>9 score) were evaluated. It is also valuable to explore the efficacy and safety of home-based Baduanjin exercise in those patients with reduced physical performance (as defined by SPPB, value 4–9).

## Ethics Statement

The Research Ethics Committee of the First Affiliated Hospital of Guangzhou Chinese Medical University has approved this study (ZYYECK[2020]045). The patients/participants provided their written informed consent to participate in this study. Written informed consent was obtained from the individual(s) for the publication of any potentially identifiable images or data included in this article.

## Author Contributions

LK, YC, and RL designed the study. YC, KC, YL, XZ, and HL wrote the manuscript. XZ, LK, and RL modified the manuscript. XZ, QC, and RL are responsible for the quality control of the test. YC, XZ, QC, LK, KC, and RL participated in the modification of the study protocol. YC and LK designed the method for statistical analyses. All authors read and approved the final version of the manuscript.

## Funding

This project is supported by the High Levels of Hospital Construction Projects of Guangzhou University of Chinese Medicine (211010010734), the Innovation and Strong Institute Project of the First Affiliated Hospital of Guangzhou University of Chinese Medicine (2019ZWB06), Science and Technology Projects in Guangzhou (202102020927), and Administration of Traditional Chinese Medicine of Guangdong Province of China (20212064).

## Conflict of Interest

The authors declare that the research was conducted in the absence of any commercial or financial relationships that could be construed as a potential conflict of interest.

## Publisher's Note

All claims expressed in this article are solely those of the authors and do not necessarily represent those of their affiliated organizations, or those of the publisher, the editors and the reviewers. Any product that may be evaluated in this article, or claim that may be made by its manufacturer, is not guaranteed or endorsed by the publisher.

## Abbreviations

AMI: Acute Myocardial Infarction
STEMI: ST-Segment Elevation Myocardial Infarction
PCI: Percutaneous Coronary Intervention
LV: Left ventricular
EF: Ejection Fraction
LVEDV: Left Ventricular End-Diastolic Volume
LVESV: Left Ventricular End-Systolic Volume
SPPB: Short Physical Performance Battery
SBP: Systolic Blood Pressure
DBP: Diastolic Blood Pressure
RPE: Rating of Perceived Exertion
ACEI: Angiotensin-Converting Enzyme Inhibitor
ARB: Angiotensin Receptor Blocker
HRQoL: Health-Related Quality of Life
UI: Utility Index
VAS: Visual Analog Score
PHQ-9: Patient Health Questionnaire-9
GAD-7: Generalized Anxiety Disorder Screener-7
CONSORT: Consolidated Standards of Reporting Trials
SD: standard deviation
AEs: Adverse Events
CRFs: Case Report Forms.
